# Establishment of the epithelial-specific transcriptome of normal and malignant human breast cells based on MPSS and array expression data

**DOI:** 10.1186/bcr1604

**Published:** 2006-10-02

**Authors:** Anita Grigoriadis, Alan Mackay, Jorge S Reis-Filho, Dawn Steele, Christian Iseli, Brian J Stevenson, C Victor Jongeneel, Haukur Valgeirsson, Kerry Fenwick, Marjan Iravani, Maria Leao, Andrew JG Simpson, Robert L Strausberg, Parmjit S Jat, Alan Ashworth, A Munro Neville, Michael J O'Hare

**Affiliations:** 1 Ludwig Institute for Cancer Research/University College London Breast Cancer Laboratory, 91 Riding House Street, London, W1W 7BS, UK; 2 The Breakthrough Breast Cancer Research Centre, Institute of Cancer Research, 237 Fulham Road, London, SW3 6JB, UK; 3 Office of Information Technology, Ludwig Institute for Cancer Research and Swiss Institute of Bioinformatics, 1015 Lausanne, Switzerland; 4 Ludwig Institute for Cancer Research, New York Branch at Memorial Sloan-Kettering Cancer Centre, New York, NY 10021, USA; 5 The J. Craig Venter Institute, 9704 Medical Center Drive, Rockville, MD 20850, USA; 6 Department of Neurodegenerative Disease, Institute of Neurology, London, WC1N 3BG, UK

## Abstract

**Introduction:**

Diverse microarray and sequencing technologies have been widely used to characterise the molecular changes in malignant epithelial cells in breast cancers. Such gene expression studies to identify markers and targets in tumour cells are, however, compromised by the cellular heterogeneity of solid breast tumours and by the lack of appropriate counterparts representing normal breast epithelial cells.

**Methods:**

Malignant neoplastic epithelial cells from primary breast cancers and luminal and myoepithelial cells isolated from normal human breast tissue were isolated by immunomagnetic separation methods. Pools of RNA from highly enriched preparations of these cell types were subjected to expression profiling using massively parallel signature sequencing (MPSS) and four different genome wide microarray platforms. Functional related transcripts of the differential tumour epithelial transcriptome were used for gene set enrichment analysis to identify enrichment of luminal and myoepithelial type genes. Clinical pathological validation of a small number of genes was performed on tissue microarrays.

**Results:**

MPSS identified 6,553 differentially expressed genes between the pool of normal luminal cells and that of primary tumours substantially enriched for epithelial cells, of which 98% were represented and 60% were confirmed by microarray profiling. Significant expression level changes between these two samples detected only by microarray technology were shown by 4,149 transcripts, resulting in a combined differential tumour epithelial transcriptome of 8,051 genes. Microarray gene signatures identified a comprehensive list of 907 and 955 transcripts whose expression differed between luminal epithelial cells and myoepithelial cells, respectively. Functional annotation and gene set enrichment analysis highlighted a group of genes related to skeletal development that were associated with the myoepithelial/basal cells and upregulated in the tumour sample. One of the most highly overexpressed genes in this category, that encoding periostin, was analysed immunohistochemically on breast cancer tissue microarrays and its expression in neoplastic cells correlated with poor outcome in a cohort of poor prognosis estrogen receptor-positive tumours.

**Conclusion:**

Using highly enriched cell populations in combination with multiplatform gene expression profiling studies, a comprehensive analysis of molecular changes between the normal and malignant breast tissue was established. This study provides a basis for the identification of novel and potentially important targets for diagnosis, prognosis and therapy in breast cancer.

## Introduction

Breast cancer is a clinically heterogeneous disease and consists of many different cell types, including normal and reactive stromal components in addition to the malignant neoplastic compartment. Moreover, it comprises a series of distinct malignant tumours that present diverse cellular features with varying differentiation status, distinct genetic changes, responses to therapy and outcome [1]. Likewise, the normal breast is also composed of different parenchymal and stromal cell types, with the terminal ductal-lobular unit being the most important feature with regard to neoplasia. The latter is composed of two morphologically recognisable cell types, epithelial cells on the luminal surface and basally located myoepithelial cells. While typical breast cancers have been traditionally regarded as exhibiting characteristics akin to luminal epithelial cells, recent data have shown that some also exhibit, in part or whole, myoepithelial/basal features [2-4]. Based on the restricted expression of genes representing the phenotypes of luminal epithelial and basal cells [4], major subtypes of breast cancer have been defined and linked to both long term survival [5] and their response to therapy [6]. Therefore, detailed characterisation of the normal luminal and myoepithelial/basal phenotypes is a prerequisite for understanding the genetic alterations that occur in breast cancers and how they may impact on disease progression and outcome.

The use of solid tissues, as in most previous breast cancer gene expression analyses, results in greatly enhanced complexity of data because of the widely varying degrees of stromal responses (desmoplasia) and inflammatory infiltrates in individual tumours. Laser capture microdissection partially alleviates this problem in respect to tumour samples, but is unsuited to the large-scale separation of the normal epithelial cell types in breast because of the close contact between these cells. Immunomagnetic separation of individual cell types from normal human breast tissue [7,8] and primary breast cancers [9] has enabled direct comparisons of normal epithelial and malignant epithelial cells to be made. Previous proteomic [9,10] and gene expression analyses of such samples [10-13] have established a partial molecular characterisation of the epithelial compartment in the normal breast and breast cancer [2], but, due to the limitations of technology available at the time of these studies, did not provide a comprehensive comparison of all proteins or transcripts.

Multiple large-scale analytical techniques now make it possible to capture entire transcriptomes of defined cell populations. Breast cancers have been extensively analysed with both expression arrays [14] and with direct sequencing techniques such as serial analysis of gene expression (SAGE) [15]. Although several studies have correlated expression data based on microarray and SAGE [16,17], a comprehensive genome-wide expression profile using a combination of complementary technologies has not yet been achieved for purified malignant epithelial breast cells in comparison with purified normal breast epithelial cells. In this study, massively parallel signature sequencing (MPSS) [18,19] and multiple genome-wide microarrays have been used to analyse immunomagnetically separated normal luminal epithelial cells and primary breast cancers substantially enriched for the neoplastic epithelial component. The aim of this study was to establish a virtually complete coverage of transcripts deregulated in the neoplastic cells of human breast cancer. In addition, expression profiles from normal luminal and myoepithelial cells have been used to identify cell-type specific transcripts and ontologically related gene sets in the differentially expressed tumour epithelial transcriptome. The use of highly enriched cell preparations in combination with a multiplatform approach to their expression analysis has revealed novel markers and potential targets, the clinical significance of some of which has also been examined, using tissue microarrays.

## Materials and methods

### Sample preparation

Ten primary cultures (approximately 10^7^) of normal human breast luminal and myoepithelial cells were prepared from reduction mammoplasty samples by double immunomagnetic sorting methods [7,8,10]. In brief, breast epithelial cells were immunomagnetically purified using combined positive magnetic activated cell sorting (MACS; Miltenyi Biotec, Auburn, CA) selection with antibodies against the luminal epithelial marker EMA (rat monoclonal ICR-2, Seralab, Leicestershire, UK) and the myoepithelial membrane antigen CD10 (mouse monoclonal CALLA clone SS2/36, DAKO Corporation, Glostrup, Denmark), followed by negative Dynabead (Dynal, UK) selection using mouse monoclonal antibodies against anti-β-4-integrin clone A9, a myoepithelial cell-surface antigen (Santa Cruz Biotechnology, CA, USA) and BerEp-4 Epithelial Antigen, a luminal antigen (DAKO Corporation, Glostrup, Denmark). Immunostaining with myoepithelial and luminal-specific lineage markers showed the final sort of epithelial cells used in this study to be >95% pure. Full details of these procedures are not only contained in previous publications [10,11], but are also appended, as required, to the Minimum information about a microarray experiment (MIAME) protocol that accompanies submission E-TABM-66 [20].

Malignant breast epithelial cells of 50 freshly isolated primary infiltrating ductal carcinomas of histological grade 2 and 3 were enriched from disaggregated tumour tissue as described previously [9]. In brief, fresh tumour biopsies (1 to 2 g) were comminuted to approximately 1 mm^3^, using scalpel blades, and subjected to a controlled disaggregation using 0.25% collagenase Type1 (Sigma-Aldrich, Dorset, UK) in L-15 medium with 2% fetal calf serum for 4 to 6 h with intermittent shaking. After brief settling, the supernatant was spun down, and the pellet resuspended in L-15 medium and passed through a 100 μm mesh filter to remove residual undisaggregated tumour fragments, plus disaggregated 'normal' organoids and ducts as well as lobules and ducts distended with ductal carcinoma *in situ*, leaving only small clusters and single cells. The latter were then reacted with the mouse monoclonal antibody F19 to fibroblast activation protein bound to sheep anti-mouse coated Dynabeads (Dynal, Paisley, UK) using the manufacturer's protocols. Almost all desmoplastic fibroblasts associated with breast cancers express this antigen strongly. Cells attached to beads were removed with a Dynal MP40 magnet; F19-negative cells were then allowed to sediment under unit gravity for 2 to 3 h (to remove most lymphocytes). The resulting preparation was then screened by phase contrast microscopy to identify those preparations in which there were few if any microvessels (the other main potential stromal contaminant not removed by fibroblast activation protein sorting), or normal tissue elements, such as ducts or acini's. Of the 50 samples, 15 were selected for this study, based on the criteria of ≥80% malignant cell content as determined by phase-contrast examination, ≥80% viability (as determined by trypan blue exclusion) and the integrity of its total RNA. The purity of both normal and malignant epithelial preparations is illustrated in Additional file 1. Informed consent to use this material for scientific research was obtained, and details of the pathology of the individual tumours are given as Additional file 2. RNA was prepared from individual samples by standard Trizol methods and pooled to give a luminal, a myoepithelial and a malignant RNA sample of >1 mg for analysis.

### MPSS analysis

MPSS was performed by Lynx Therapeutics, (CA, USA) according to the Megaclone 'signature' protocol [18,19]. Briefly for each library synthesis, after DNase treatment of approximately 300 μg total RNA from normal luminal and malignant breast epithelial pools, cDNA was generated from poly(A)+ RNA, and amplified copies of each cDNA clone were attached to beads. The sequence adjacent to the poly(A) proximal *Dpn*II site was determined by cycles of ligations to fluorescently tagged 'decoding' oligonucleotides and cleavages by restriction enzymes. Each sequence signature comprises the *Dpn*II restriction recognition site (GATC) and 13 contiguous nucleotides. The raw data resulted from four sequencing runs, collected in two reading frames offset by two nucleotides relative to the anchoring restriction enzyme site and generating approximately 2 to 3 × 10^6 ^sequences. Signatures that were seen in at least two independent runs (reproducible) and were present at a frequency of more than three transcripts per million (tpm) in one sample (significant) were selected for further analysis.

As a basis for the matching of signature sequences to transcripts, we used our own reconstitution of the human transcriptome database (HTR) [21-23] based on a comprehensive set of cDNA to genome alignments that are merged into gene models representing the detailed structure of human transcribed regions. Each HTR contains a cluster of cDNA sequences, similarly to the NCBI/UniGene database. The annotation of the signature was then performed in two steps as described previously [22], using the NCBI35 assembly of the human genome. Firstly, a 'signature-centric' annotation was performed, where sequence signatures were mapped to either one or more transcribed regions of the genome, including repetitive sequences, ribosomal, mitochondrial and non-mapped transcripts. In the second step, only signatures from the 'signature-centric' annotation that matched exactly or had one nucleotide mismatch to known transcribed regions were retained to form the 'gene-centric' version. When different sequence signatures mapped to the same gene, counts were combined. To identify genes with significant differences (*P *value ≤ 0.05) in representation in the two RNA pools, the absolute difference in abundance between the malignant and the normal epithelial RNA sample was determined and log_2 _transformed, resulting in a relative expression measurement.

### Microarray analysis

The same total RNA pools were hybridised onto a 20 k cDNA microarray (20 k brk, constructed at The Breakthrough Breast Cancer Research Centre, Institute of Cancer Research, London, UK containing 19,391 sequence-validated IMAGE clones), Affymetrix Human Genome U133 Plus 2.0 GeneChip (Affymetrix, Inc., Santa Clara, CA, USA), CodeLink™ Human Whole Genome Bioarray (GE, Healthcare, formerly Amersham Biosciences, Chandler, AZ, USA) and Agilent Whole Human Genome Oligo Microarray 44 k cDNA array (Agilent Technologies, Palo Alto, CA, USA). Three technical replicates of each RNA pool were amplified, labelled and hybridised according to manufacturer's guidelines. Where necessary an RNA pool consisting of breast cancer cell lines was used as a reference sample [11] and dye-swap hybridisations were performed. All primary array data are available through ArrayExpress [20]; they comply with MIAME standards, with the accession number E-TABM-66. Overlay of each microarray platform with MPSS was done by mapping the sequence information of probes and probe sets to the same HTR database as used for MPSS tag mapping (see above). Only those microarray features that were unambiguously mapped to a single HTR cluster were included for further studies. All preprocessing of each microarray platform and further statistical analysis was performed in the R 2.1.1 environment [24] by making extensive usage of the limma package [25] in BioConductor 1.6 [26]. For the Affymetrix platform, probe-level data were normalised and expression data were summarised by the robust multi-array analysis [27]; cyclic lowess normalisation was applied to the CodeLink™ expression data through the *codelink *0.7.2 package in R 2.3; for the Agilent microarrays, global normalisation with no background correction was applied; and for the 20 k brk microarrays, raw expression data were print-tip normalised and background corrected. Relative measurements for each transcript were given as a log_2 _fold ratio, and only genes with a false discovery prediction of *P *≤ 0.05 were regarded as significantly differentially expressed when using Benjamini and Hochberg's *P *values adjustment [28].

### Gene Ontology

Genes were categorised with respect to their biological process, cellular role, molecular function, using Onto-Express (OE) [29,30]. The most significant perturbed biological processes were determined with respect to the number of genes expected for each Gene Ontology (GO) category based on their representation on the Affymetrix U133 Plus 2.0 array. Statistical significance was determined by using OE's hypergeometric probability distribution and Bonferroni correction options, and annotations with *P *≤ 0.05 were accepted as significant. Gene set enrichment analysis (GSEA) comparing luminal and myoepithelial gene signatures was done using described methods [31]. Biological processes were ranked according to their significance of enrichment, and the validation mode measure of significance was used to identify those of greatest enrichment.

### Semiquantitative RT-PCR

Total RNA (10 μg) from the normal luminal epithelial and the malignant epithelial RNA pool was used for each 40 μl reverse-transcription reaction, and 10 μl of 1/50 diluted cDNA was used per 30 μl PCR. RT-PCR was performed by using the Applied Biosystems AmpliTaq Gold, Cheshire, UK, with either 25 or 30 cycles, each consisting of 30 s at 94°C, 30 s at 55°C, and 45 s at 72°C. PCR products were visualised on 2% Invitrogen agarose E-Gels 96 Gels (Invitrogen Life Technologies, Carlsbad, CA, USA).

### Immunohistochemistry and tissue microarray analysis

A cohort of invasive breast carcinomas from 245 patients treated with surgery (wide local excision or mastectomy) and adjuvant anthracycline-based chemotherapy was retrieved from the Department of Histopathology files of the Royal Marsden Hospital (London, UK) with appropriate local Ethical Committee approval. Representative blocks were reviewed by a pathologist (JSRF) and selected cores were incorporated in two duplicate tissue microarray (TMA) blocks [32,33]. Full details of the TMA are given as Additional file 3. TMA samples were dewaxed in xylene, cleared in absolute ethanol and blocked in methanol for 10 minutes. Antigen retrieval for cartilage oligomeric matrix protein (COMP) and IL8 was by boiling slides in citrate buffer (pH 6) for 2 minutes in a pressure cooker, after which they were blocked with normal horse serum (2.5% for 20 minutes; Vector Laboratories VL, Burlingame, CA, USA) and endogenous biotin blocked by pre-incubating with avidin (15 minutes) and biotin (15 minutes). They were then incubated with anti-COMP antibody (1/50; Serotec, Oxford, UK) or IL8 antibody (1/5; Serotec) for 1 h at room temperature. For immunohistochemistry of Periostin (POSTN), sections were pretreated by microwaving in Dakocytomation (Glostrup, Denmark) pH 6 antigen retrieval buffer for 18 minutes, blocked, and anti-POSTN antibody (1/1500; Biovendor Laboratory, Heidelberg, Germany) applied for 30 minutes at room temperature. Antibody binding was detected using Vectastain Universal ABC (VL), visualised with 3,3'-diaminobenzidine DAKO (Corporation, Glostrup, Denmark). Full details on the distribution of ER, PR, HER2, EGFR, CK 14, CK 5/6, and CK 17, as well as P53 (DO7, 1/200; DAKO Corporation) are described elsewhere [33] and summarised in Additional file 3. To evaluate the proliferative activity of tumour cells, immunohistochemical detection of MIB1 antibody to detect Ki-67 nuclear antigen (1/300; DAKO Corporation), which is associated with cell proliferation, was carried out under the same conditions [33]. For these markers, only nuclear staining was considered specific. Ki67 (MIB1) staining was scored low if less than 10% of neoplastic cells were positive, intermediate if 10% to 30% of neoplastic cells were positive and high if more than 30% of neoplastic cells were positive [32]. Tumours were scored positive for P53 if >10% of the nuclei of neoplastic cells displayed strong staining [32].

Cumulative survival probabilities were calculated using the Kaplan-Meier method/log-rank test. Differences between disease-free interval and survival were tested with the log-rank test (two-tailed, confidence interval 95%) using the statistical software Statview 5.0., NC, USA. Multivariate analysis was performed using the Cox multiple hazards model. A *P *value < 0.05 in the univariate survival analysis was used as the limit for inclusion in the multivariate model.

## Results

### MPSS analysis of normal luminal and malignant breast cancer cells

The gene expression profiles that were obtained by MPSS analysis yielded 24,288 and 28,404 signature sequences for the malignant and the normal breast epithelium, respectively; these were pared down to the 'signature-centric' version containing 14,245 uniquely mapped and expressed transcripts for the malignant sample and 10,249 transcripts for the normal luminal epithelial sample (Table 1). Based on our HTR (described in Materials and methods [21]), these transcripts corresponded to 8,421 and 6,477 HTR clusters in the malignant and the normal RNA samples, respectively (Table 1), of which 3,191 genes were uniquely expressed in the malignant sample, and 1,297 in the normal sample. To define differential expression, a comparative Poisson test was applied [34] and 6,553 genes were identified that showed a differential expression measurement with *P *≤ 0.05. (Raw and annotated MPSS data are provided as Additional file 4) Expression levels of differentially upregulated transcripts in the tumour sample ranged from less than 10 tpm (*ESR1*, *EGF*, *GPR150*, *GADD45BGIP1*), to over 1,000 tpm (*COL1A1*, *SCGB2A2*, *SELE*, *IL8*).

### Establishing a microarray validated transcriptome

The MPSS derived transcriptomes were compared with gene expression profiles of the same RNA pools obtained using three oligonucleotide genome-wide microarrays, Affymetrix U133 Plus 2.0 GeneChip and CodeLink™ Human Whole Genome Bioarray, Agilent Whole Human Genome Oligo Microarray 44 k cDNA array and 20 k brk cDNA microarray. These different microarray platforms were chosen to achieve the highest possible coverage of known transcribed sequences. Features from all platforms were mapped to HTR clusters and our analysis was restricted to those that mapped unambiguously to one HTR cluster. For the Affymetrix platform 41,322 of 54,613 (75.66%) features could be assigned to unique HTR clusters; for CodeLink™ 28,949 of 54,841 (52.78%); for Agilent 32,402 of 44,290 (73.15%); and for the 20 k brk 12,055 of 19,959 (60.4%). Overlay of the transcript coverage of each microarray demonstrated that each platform contributed a set of unique genes as well as those common to other platforms, justifying the use of more than one microarray platform. (Full annotation of each microarray platform to HTR clusters is available as Additional file 5) The microarray features of all four platforms provided a total coverage of 26,103 HTRs, and 6,342 out of 6,553 (96.8%) of the differentially expressed transcripts obtained by MPSS were represented on one or more of these genome-wide platforms.

**Figure 1:**
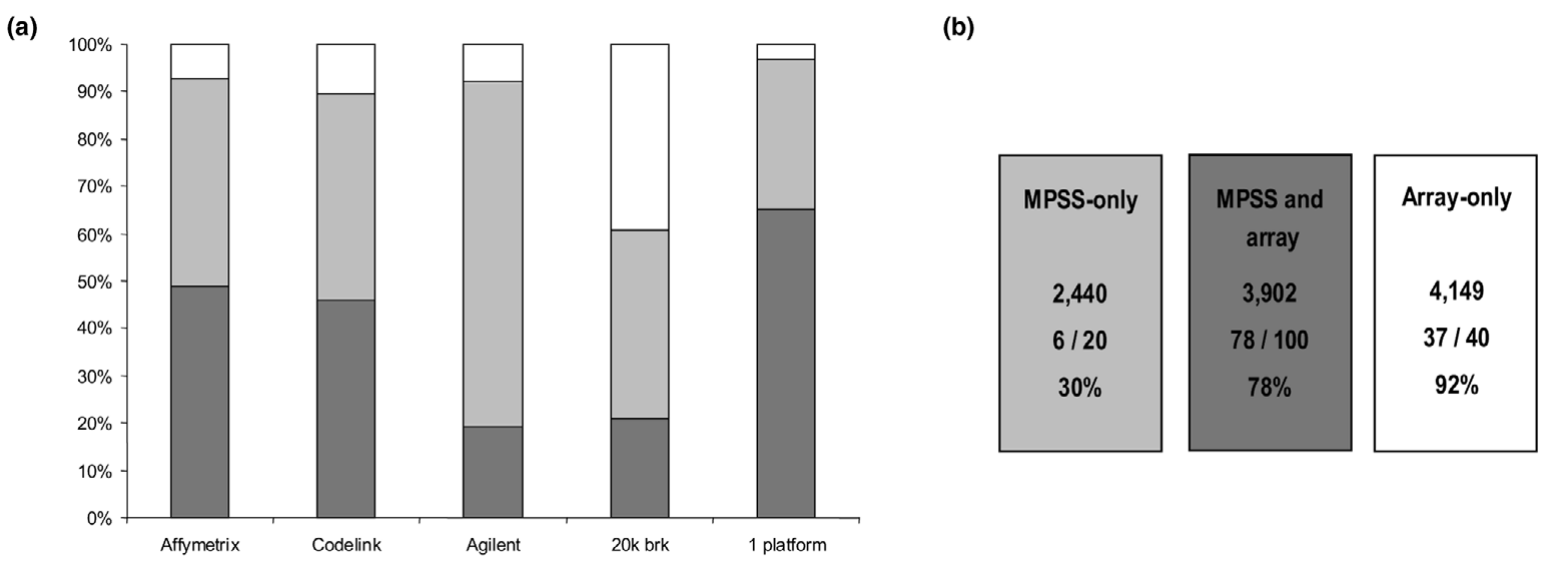
Comparison of massively parallel signature sequencing (MPSS) data with microarray analysis. Differentially expressed gene profiles from MPSS (100%) were overlaid with each microarray platform individually. **(a) **Percentage of coverage (light grey) and concordance in differential expression between MPSS and individual arrays (dark grey) are shown together with the combined coverage and confirmation by at least one array (1 platform). **(b) **Enumeration of the differentially expressed transcripts detected by "MPSS-only", by "MPSS and array", and those transcripts reported as differential by at least two arrays, but not by MPSS ("Array only"). The results obtained by RT-PCR for these subgroups are shown below (see Additional file 6).

Having established a common denominator in terms of gene annotation, those genes reported as differential between the normal and malignant tumour sample by microarrays were defined and then compared with the MPSS data. The criteria for differential expression used were that expression measurements between the normal and the malignant sample reported had to be both statistically significant (*P *≤ 0.05) and in the same direction (up or down). Out of the four microarray platforms, the two single colour oligonucleotide platforms (Affymetrix and CodeLink™) validated as differential 3,206 (48.9%) and 3,004 (45.8%) of all MPSS transcripts present on their platforms, respectively, whereas the two-colour microarray technologies confirmed only 1,257 (19.1%) and 1,379 (21%), for Agilent and 20 k brk, respectively (Figure 1a). Overall, a total of 3,902 genes were obtained in which at least one microarray confirmed the MPSS data without any other platform reporting an opposite result (Figure 1a; 1 platform). Expression measurements for 2,440 MPSS differential transcripts could not be confirmed using any of these microarray platforms (Figure 1b, "MPSS-only"). The microarray data were also used to identify any genes reported as differential by at least two platforms, but which did not appear as such in the MPSS analysis. This comprised a total of 4,149 transcripts (Figure 1b, "Array-only"). To establish which of those sets could be most relied on to constitute the validated differential tumour epithelial transcriptome (DTET), examples of each group were analysed by semi-quantitative RT-PCR (Figure 1b). This showed that only 30% (6/20) of the "MPSS-only" identified differentials could be validated, while 78% (78/100) and 92% (37/40) of the "MPSS and array" and "Array-only" differentially expressed transcripts were reported as differential by RT-PCR (Additional file 6). The comparison of RT-PCR results was not given any statistical treatment and is simply presented to illustrate that the array confirmed differentials have a much lower false positive rate (20% to 70%). Consequently, the latter two groups were combined and comprised 8,051 up- and down-regulated genes that constitute the DTET and were subjected to further analysis (Additional file 7).

### Functional classification of differentially expressed genes

**Figure 2:**
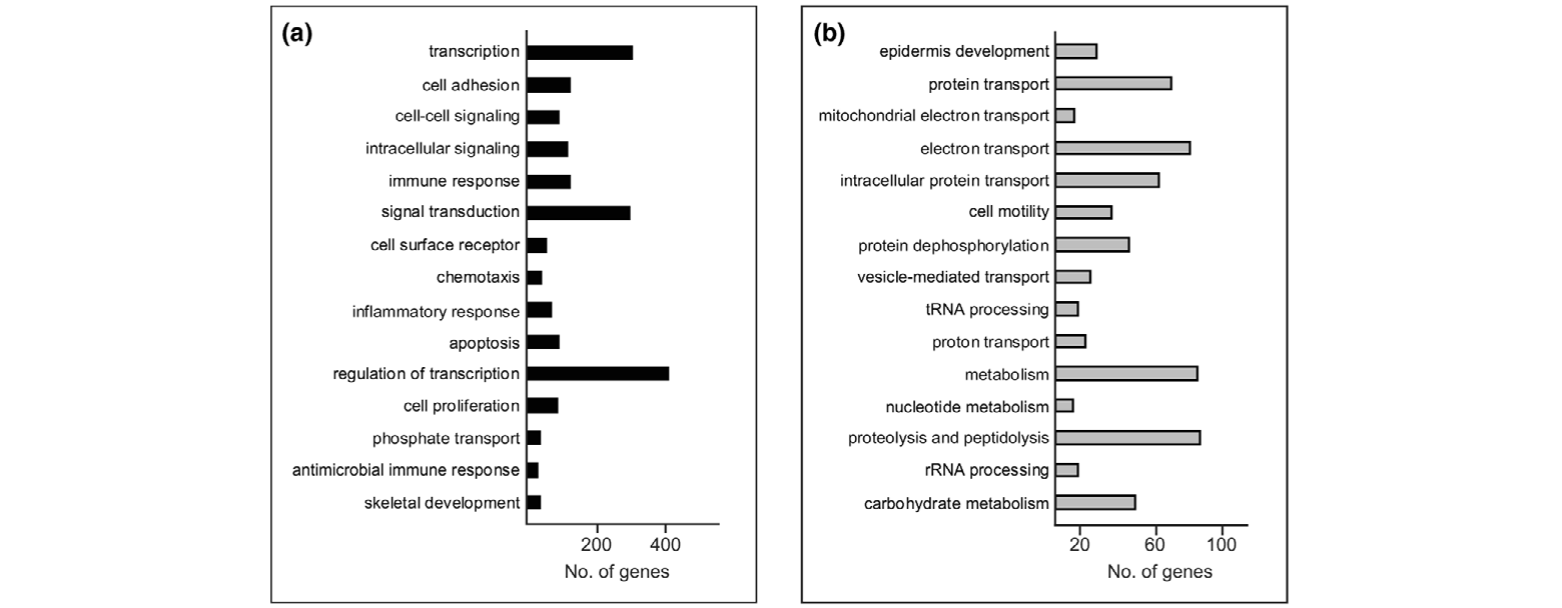
Functional classification of the differentially expressed epithelial tumour transcriptome. The top 15 biological processes showing overall **(a) **up-regulation and **(b) **down-regulation are shown. The biological processes are ranked from top to bottom according to their ascending *P *value as described in the Materials and methods. The numbers of genes within each process that are up-regulated or down-regulated for each category are also shown as black and grey bars, respectively.

GO classification of the 8,051 genes of the DTET revealed that, as might be expected, multiple cellular processes, such as transcription, signal transduction, cell adhesion, cell cycle, metabolism, transport and development, are different in normal luminal epithelium and their malignant counterparts (the full list of perturbed biological processes is provided as Additional file 8). In terms of overall differences, the largest functional group of up-regulated transcripts (Figure 2a) corresponded to genes associated with transcription and regulation in transcription, in agreement with several other profiling studies. The second largest functional group comprised genes involved in signal transduction. These consisted, amongst others, of genes encoding proteins involved in mitogen-activated protein kinases (MAPK) signalling (*FGF4*, -7, -13, *IL1A*, *IL1B*, *NGFB*, *TGFB1 *and *TGFB3*) and the JAK-STAT signalling pathway (*IL6*, *IL10*, *OSM*, *SPRY2*), as well as ligands and receptors involved in cytokine-cytokine interaction, including members of the CXC and CC chemokines, platelet-derived growth factor, gp130, tumour necrosis factor and transforming growth factor-β subfamilies. Many of these genes have already been correlated with breast cancer growth and invasion, and their epithelial expression has been demonstrated. In contrast to previously published SAGE data, comparing purified normal breast epithelial tissue with solid tumour breast tissue [12,13] in which reduced expression of cytokines such as IL6 and IL8 was observed, higher abundance of these genes was detected in our malignant breast epithelial sample in comparison with the normal luminal sample. Ninety genes belonging to the GO category of 'apoptosis', including members of the BAG family (*BAG1*, *BAG2*, *BAG3*), as well as members of the breast cancer 'proliferation signatures' (*BUB1*, *PLK1*, *CCNE1*, *CCND1 *and *CCNB1*) were also identified as up-regulated in our DTET [35,36].

The most significantly perturbed functional gene set identified in the down-regulated tumour epithelial transcriptome (Figure 2b) was epidermis development, including members of the kallikrein family (*KLK5*, *KLK7*, *KLK8*, *KLK10*) and the keratin family (*CK10*, *CK14*), as well as the family of extracellular matrix glycoproteins, such as *LAMC2*, *LAMB3 *and *LAMA3*. The second most perturbed subset of down-regulated genes included several members of the RAS-related proteins, *RAP1A*, *RALB*, *RAB5B*, *RAB4A*, *RAB3B*, *RAB2 *and *RAB25 *(protein transport; Figure 2b), some of which counteract the mitogenic function of RAS-MAPK signalling pathways [37].

### Differentially expressed transcripts in normal breast epithelial cells

**Figure 3:**
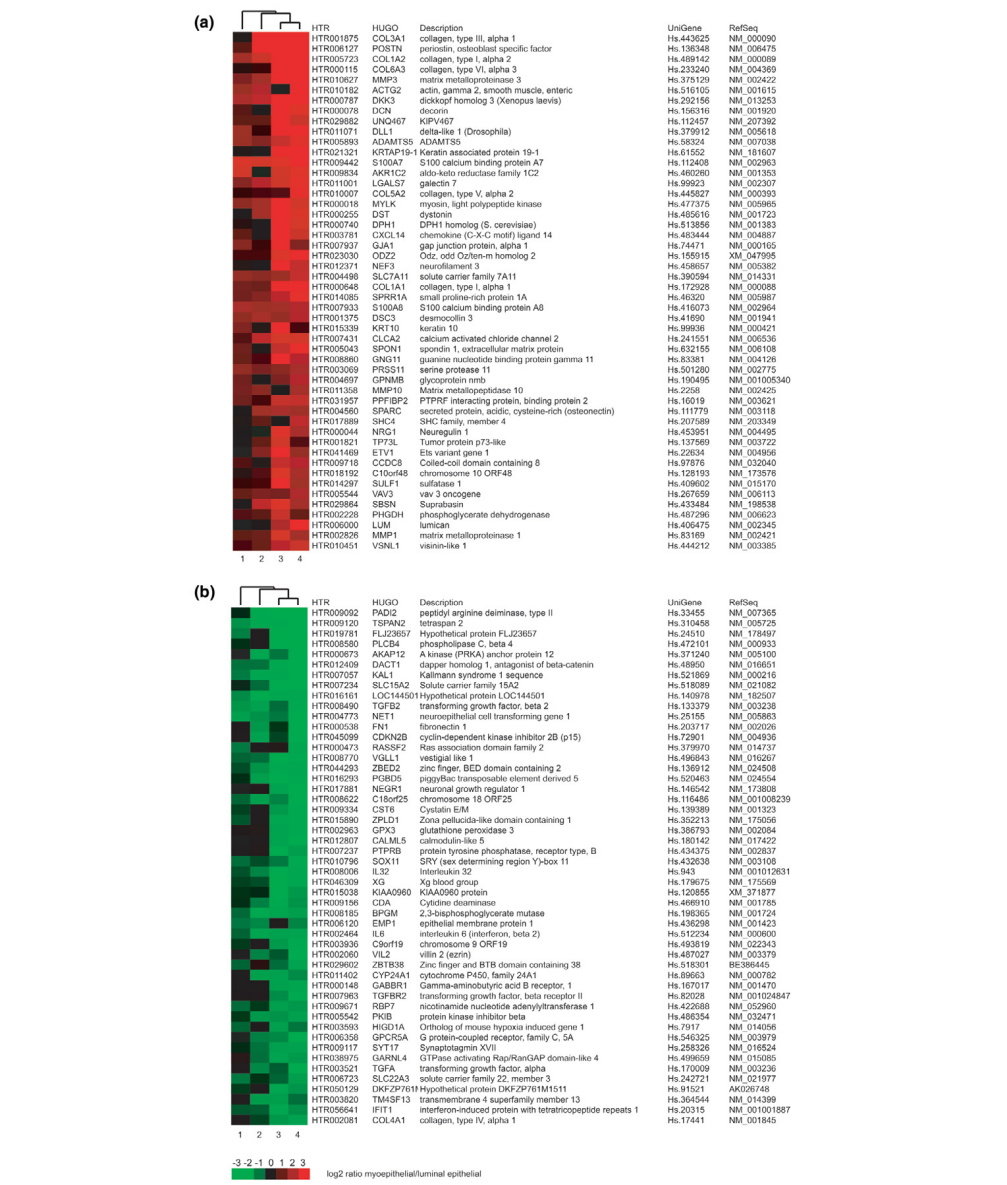
Heatmap of the top 50 genes from the luminal-specific and myoepithelial-specific transcriptomes. Genes were ranked in order of fold change (myoepithelial over luminal) for each platform separately after which a median rank over all four platforms was determined. Genes are listed with their human transcriptome database (HTR) cluster, HUGO Name, description and UniGene and RefSeq identifiers. Green corresponds to luminal-type; red to myoepithelial-type; black indicates no corresponding microarray feature. Expression measurements obtained by: 1, Agilent; 2, 20 k brk; 3, CodeLink; 4, Affymetrix platform.

Whether tumours exhibit a luminal or myoepithelial/basal phenotype has been correlated with prediction and prognosis in breast cancer [2-4]. Global transcriptomes of normal myoepithelial and luminal epithelial cells were, therefore, compared to identify all transcripts that were differentially expressed in these normal cell types. The purpose was to further define breast epithelial specificity within the tumour transcriptome by annotating the DTET with respect to their expression in these normal epithelial cell types. Differential gene expression profiles of immunomagnetically purified luminal and myoepithelial cell samples were established using the criterion of differential detection by at least two of the four genome-wide microarray platforms, as used previously when comparing the normal luminal with the malignant sample. We identified 907 transcripts with higher abundance in the normal luminal cells and 955 transcripts were higher in the normal myoepithelial cells. These collectively comprised the differential normal epithelial transcriptome. The top 50 discriminator genes over all four microarray platforms are shown in Figure 3 (complete list is given as Additional file 9). These genome-wide gene signatures agreed with previous data from individual luminal and myoepithelial sample analyses [11]. All the main classifiers for the myoepithelial cell type, such as *LGALS7*, *S100A2*, *SFN*, *SPARC *and *CAV1 *(and *CD24*, *LCN2*, *CLDN4*, *MUC1 *and *SEMA3B *for the luminal epithelial cell type) were identified as differential in the present study. However, as expected from the enhanced coverage provided by the methods used here, many other genes that may play an important role in the biology of these two cell types were also identified (for example, *PADI2*, *TSPAN2*, *DACT1 f*or the luminal, and *POSTN*, *DCN*, *ADAMTS5 *for the myoepithelial cell type).

### Enrichment of luminal and myoepithelial genes and gene sets in the differential tumour epithelial transcriptome

**Figure 4:**
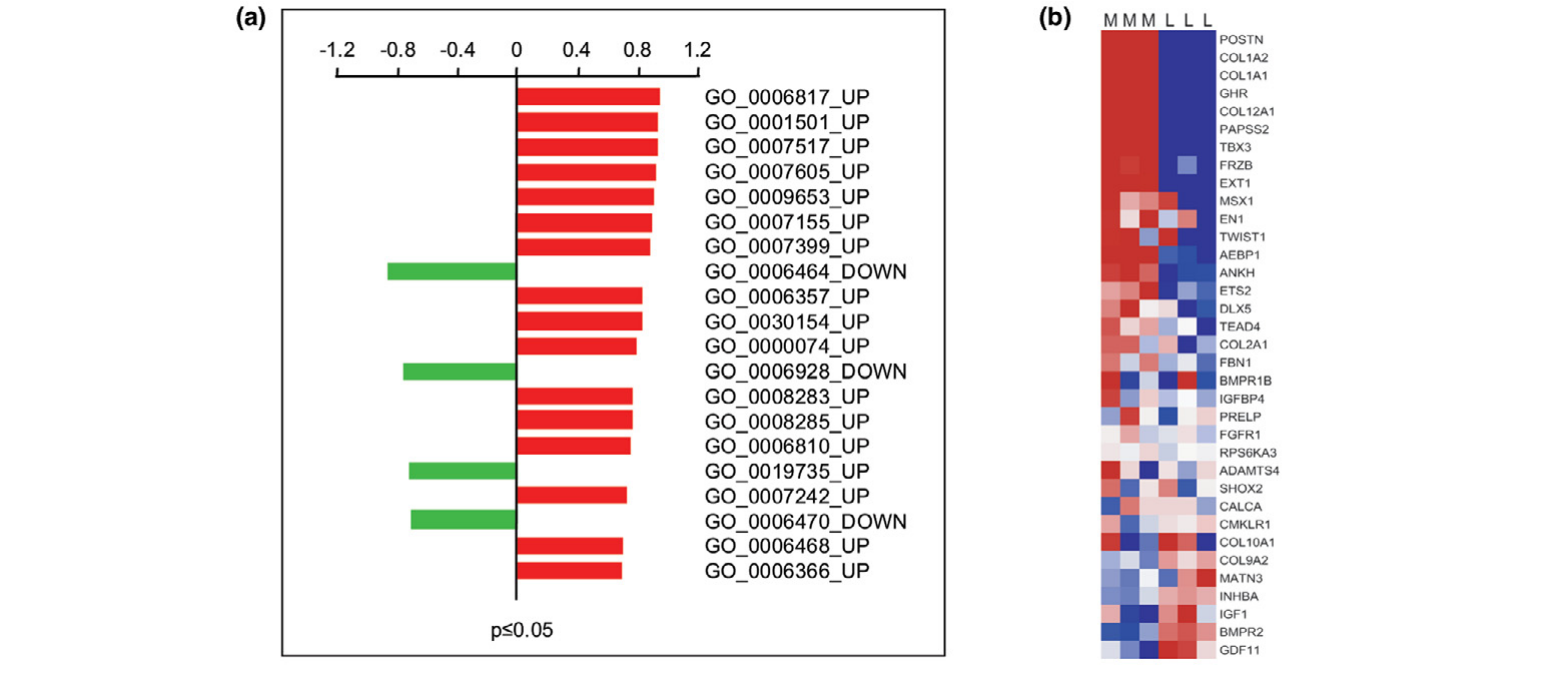
Enrichment of luminal and myoepithelial transcripts in the differentially expressed epithelial tumour transcriptome. **(a) **The top 20 deregulated biological processes identified by gene set enrichment analysis that are enriched in luminal (green) and myoepithelial (red) expression are shown. The definition of each Gene Ontology (GO) category is given in Additional file 8. **(b) **Heatmap of the skeletal developmental gene subset (GO:0001501) based on the Affymetrix expression data. L (luminal) and M (myoepithelial) show results from individual arrays. Genes are ranked according to their significance of enrichment as described in the Materials and methods.

To identify functionally related gene sets of luminal or myoepithelial phenotype within the DTET, GSEA was carried out on the perturbed biological processes that were statistically significant (*P *≤ 0.05) and composed of at least 10 genes [31]. This resulted in a total of 72 gene sets, 53 and 19 for the up- and down-regulated modules, respectively (Additional file 8). In the top 20, four categories showed enrichment of genes belonging to our luminal transcriptome, including protein modification (GO:0006464), cell motility (GO:0006928) and protein dephosphorylation (GO:0006470), as down-regulated modules, as well as antimicrobial immune response (GO:0019735) as an up-regulated one (Figure 4a). Overall, GSEA analysis showed marked enrichment for the expression of myoepithelial genes in the functional groups of the tumour overexpressed transcripts compared to the luminal epithelial transcriptome (Figure 4a). The gene set with the most statistically significant representation of myoepithelial type genes consisted of members of the collagenase family (GO:0006817), with *COL3A1*, *COL6A1*, *COL1A1*, *COL5A12*, *COL15A2*, *COL1A1 *and *COL12A1 *representing the discriminator genes. The second most statistically significant enrichment of expression in myoepithelial type genes with higher abundance in the malignant breast epithelium was found in the functional category of skeletal development (GO:0001501; Figure 4a,b). This set of bone related genes included *COL1A2*, *COL1A1*, *GHR*, *COL12A1*, *PAPSS2*, *TBX3*, *FRZB*, *EXT1*, *MSX1*, *EN1*, *TWIST1 *and *AEBP1*, with *POSTN *being the most prominent discriminator of this gene set (Figure 4b).

### Clinical significance of POSTN using tissue microarray analysis

**Figure 5:**
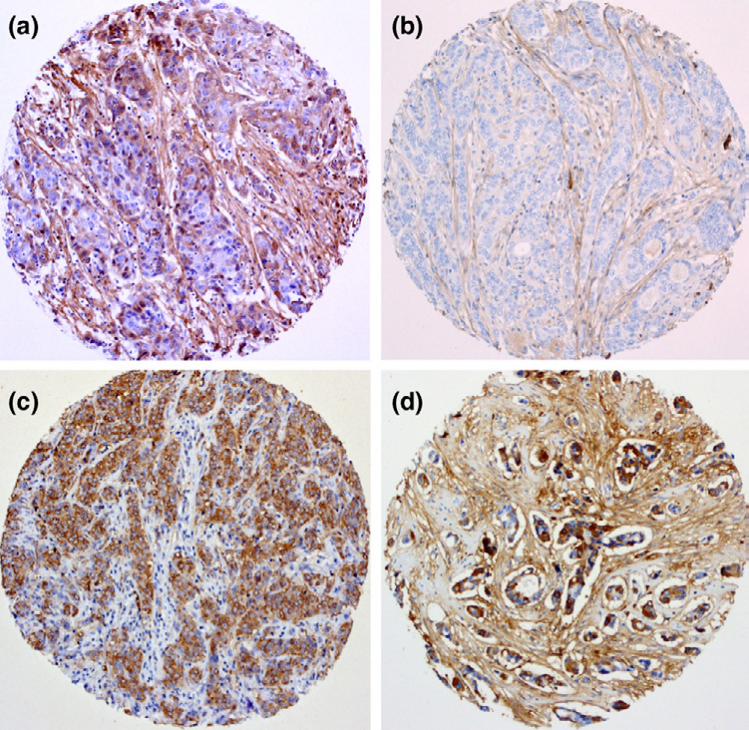
Immunohistochemical analysis of periostin (POSTN), IL8 and cartilage oligomeric matrix protein (COMP). **(a) **POSTN-positive invasive ductal carcinoma (IDC; ×400), in which both epithelial and stromal cells show cytoplasmic expression. **(b) **POSTN-negative IDC in which only the spindle shaped stromal cells are stained (×400). **(c) **IL8 (×100), showing positive staining only in the malignant breast epithelial cells. **(d) **COMP expression in the epithelial and stromal cells of an IDC, showing strong expression in both stromal and epithelial cells (×100).

**Figure 6:**
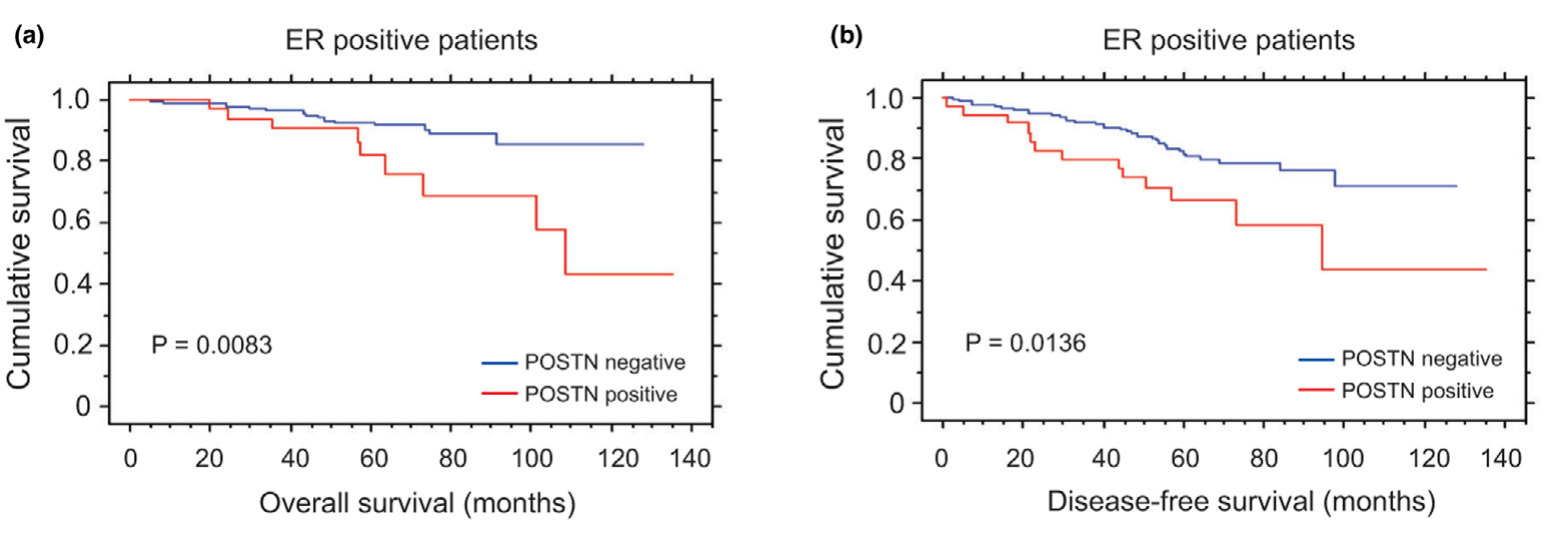
Cumulative Kaplan-Meier curves for epithelial expression of periostin (POSTN). A cohort of poor-prognosis estrogen receptor (ER)-positive tumours was analysed showing: **(a) **a significantly shorter overall survival (*P *= 0.0083); **(b) **a shorter disease free survival (*P *= 0.0136).

To evaluate whether the luminal and myoepithelial annotations of our epithelial deregulated transcriptome identify genes with any correlation with clinical outcome in breast cancer, we performed immunohistochemical analysis POSTN on a tissue microarray consisting of 245 primary breast tumours. POSTN, usually expressed in mesenchymal cells, was chosen, not only because it was one of the most highly differentially expressed genes in normal myoepithelial cells over all microarray platforms (Figure 3), but also because it belongs to the functional group of skeletal development that showed overall myoepithelial-specificity and up-regulation in the malignant breast epithelium (Figure 4b). When POSTN expression was examined at the protein level, no detectable expression was observed in the normal breast epithelium, but only in the stroma, in concordance with its known mesenchymal expression (not shown). However, 42/224 (18.75%) invasive breast carcinomas clearly showed epithelial expression (Figure 5a), whereas the remainder showed the expected expression pattern only in the stroma (Figure 5b). POSTN expression in neoplastic cells was significantly correlated with positivity for progesterone receptor (PR) (*P *< 0.05) and low proliferation rates as defined by Ki67 (MIB1) staining (*P *< 0.05) (Additional file 10). When the whole cohort was analysed, POSTN-positive breast cancers showed a trend towards a poorer outcome, although this did not reach statistical significance (Additional file 11a,b). Since the estrogen receptor (ER) status is the most important marker in defining the prognosis and treatment of breast cancer, the correlation of POSTN expression with overall survival and disease free survival was analysed in ER-positive and ER-negative subgroups. No significant correlation was observed in the ER-negative cohort. However, within the ER-positive subgroup, 20.8% (37/178) of breast tumours were positive and there was a significant correlation with both overall survival (*P *= 0.0083) and disease-free survival (*P *= 0.0136) (Figure 6a,b, respectively). In this cohort, modified Bloom-Richardson grade (*P *< 0.01), lymph node status at diagnosis (*P *< 0.005) and POSTN expression (*P *< 0.05) were statistically significant predictors of disease-free survival in univariate analysis, whereas only lymph node status at diagnosis (*P *< 0.001) and POSTN expression (*P *< 0.01) were associated with overall survival in univariate analysis. By multivariate analysis of disease-free survival in the ER-positive cohort, POSTN did not reach formal statistical significance as an independent factor (*P *= 0.0833) (Table 2, italics), although it did constitute an independent prognostic factor for overall survival (*P *= 0.0168) (Table 2, bold). Two other genes that showed up-regulation in the malignant breast epithelium were also analysed on the protein level by tissue microarray, namely those encoding COMP [38], a skeletal developmental protein that was not differentially expressed between luminal and myoepithelial cells, and IL8, an inducer of bone resorption. Similarly to POSTN, COMP and IL8 could be clearly detected in the epithelial cells of 21% and 13.9% invasive breast carcinomas, respectively (Figure 5c,d). In contrast to POSTN, however, there was no correlation of COMP or IL8 tumour staining with age, grade, stage, ER, PR, disease-free interval or overall survival, although epithelial expression of the mesenchymal markers POSTN and COMP correlated significantly with each other (Additional file 10).

## Discussion

Using highly enriched populations of malignant breast epithelial cells and normal epithelial cells, obtained from immunomagnetic cell sorting, we have established genome-wide molecular signatures specific to the epithelial compartments of both the normal and the malignant human breast. Combining gene profiles obtained from different expression platforms, including direct high-throughput sequencing (MPSS) and multiple microarray platforms, yielded a validated transcriptome comprising 8,051 differential transcripts. These data provide a basis for the molecular changes that occur in the transition from normal luminal to malignant epithelial cells, and also allow further analysis of solid breast tumour (neoplastic plus stroma) gene expression studies, enabling those genes of specific epithelial origin to be identified in respect to progression, prediction of outcome and metastasis. The expression data obtained from the normal luminal and myoepithelial cells have extended our previous analysis of these normal cell types [11], and provide gene sets that can be used to comprehensively specify the epithelial phenotype expressed in breast tumours, as well as defining new markers of each cell type.

The data presented here report for the first time the application and validation of the MPSS sequencing technology to malignant human breast epithelial cells and their normal counterparts. MPSS expression studies of different human cell lines and normal tissues have already shown that this technology represents the most comprehensive sequencing methodology available at present, in terms of gene coverage and quantitative assessment of gene expression [22,39]. With over 10^6 ^sequencing reactions per sample [18,19], it is comparable in scope with the now commonly used genome-wide microarray profiling methods, as also used in the present study. Comparative studies of genome wide data sets are entirely dependent on the choice of common denominator for annotation [40]. By using our sequence based mapping, 97% of MPSS tags could be aligned with individual features on genome-wide microarrays, indicating that the vast majority of the expressed sequence tags in the normal and malignant breast epithelium MPSS libraries represent known transcripts, in agreement with the recent data suggesting that MPSS identifies very few truly novel genes [39]. Given the significant methodological differences between microarray and MPSS analysis, the fact that more than 65% of our MPSS differential data set showed concordance with expression profiling obtained by several different microarray platforms, represents a good overlap compared with other examples of sequence versus array data [41]. However, a substantial number of differentially expressed genes (4,149) measured on at least two microarray platforms were not identified as such by MPSS, and a significant number of MPSS differential transcripts (2,440) were not confirmed on any array (Figure 1), implying a relatively high false positive and false negative rate of the MPSS methodology. This probably reflects the known limitations of the MPSS technology [39], particularly with regards to transcripts that were not detected (zero counts) in one sample, as well as genes lacking appropriate restriction enzyme sites required for this technology. However, individual microarray platforms themselves differ substantially [42] and a multiplatform approach, as used here, clearly defines a robust DTET seen by every technology.

Another important feature of our DTET is the use of purified epithelial cells, derived by both positive and negative immunomagnetic sorting in which the contamination of malignant samples with stromal cells is reduced to a minimum, and normal luminal and myoepithelial cells are separated from short-term primary cultures. Although the profiling techniques used represent the global transcriptomes of purified normal and neoplastic breast epithelial cells in highly enriched preparations, it is conceivable that even a small contamination of the malignant samples by normal or reactive stromal cells, as well as the induction of inflammatory genes due to *in vitro *manipulation, could result in false positives. However, verification of the probable epithelial origin of differentially expressed genes can be obtained by comparing expression data from breast epithelial cell lines [22], breast tumour cell lines or, as in the present study, by immunohistochemistry, all of which show that, for example, IL8, is a *bona fide *epithelial tumour-associated product [43,44]. One of the features of normal luminal epithelial cultures is the loss of estrogen receptor expression [45]. The microarray gene expression profiling currently used to classify breast cancers supports the paradigm that ER status is the most important phenotype in breast cancer and has led to the classification of breast cancers into luminal A (ER-positive good prognosis) and luminal B (ER-positive poor prognosis), and ER-negative myoepithelial/basal and HER2 subtypes, each with distinct differences in prognosis and response to therapy [4,5,46]. Genes identified in this study representing the normal luminal epithelial phenotype are distinct from the subset of genes that are associated with ER expression and are used to classify 'luminal' breast tumours. Thus, we are able to define the luminal phenotype independently of ER status. In contrast, our myoepithelial signature contains several members of the previously reported gene clusters identifying basal-like breast cancers. Some of these have been previously identified as myoepithelial genes in the normal breast epithelium, for example, *TIMP3*, *SPARC*, *JAG1*, *PRSS11 *and *CAV-1 *[11], and some of them, such as *S100A7*, *SPARC *and *CNN1*, have previously been shown individually to be correlated to poor outcome [5,11,47]. Since our cell type specific gene signatures were derived from phenotypically well characterised cell types compared to empirical stratification based on expression data, we were also able to identify a range of myoepithelial type genes in ER-positive tumours as well as those in basal-like breast cancers. Thus, although the majority of the primary breast tumours within our malignant pool were ER-positive 'luminal' tumours, a significant number of up-regulated gene sets also showed myoepithelial expression. The observation of myoepithelial genes such as *SFRP2*, *DCN*, *POSTN*, *LUM*, *COL1A2 *and *COL11A1*, which showed higher expression in ER-positive compared to ER-negative breast tumours in two other breast cancer tumour profiling studies [48,49], proved the value of such an approach and demonstrated the heterogeneity of breast tumours with respect to the levels of luminal epithelial and myoepithelial gene expression. The potential clinical significance of the expression of myoepithelial/basal genes in ER-positive tumours has been highlighted by recent data showing that the promoter DNA methylation of the classic myoepithelial marker *S100A2 *is correlated with a poor prognosis in ER-positive tumours [50]. In contrast, increased levels of expression of phosphoserine aminotransferase (encoded by *PSAT1*), which was another gene also identified in our myoepithelial transcriptome, was the strongest predictive marker for a poor response to tamoxifen therapy in ER-positive tumours [50]. Our observation that the malignant epithelial expression of POSTN, also a myoepithelial/basal gene, is associated with poorer survival (*P *= 0.0083) in ER-positive tumours demonstrates that the normal epithelial annotation of tumour transcripts can identify many other types of myoepithelial/basal genes, including those associated with a poor outcome.

An important question is whether the expression of myoepithelial/basal genes in breast cancers are responsible for the prognosis and poor response to therapy or are merely surrogate markers thereof. There are several lines of evidence to suggest that POSTN may play a role in the biology of breast cancer [51,52]. POSTN is a ligand of α_v_β_3 _integrins and promotes adhesion and migration of epithelial cells [51]. Clinical studies of periostin expression in human cancers have demonstrated that increased expression of POSTN is correlated with tumour angiogenesis and metastasis [52-54]. In primary breast tumours, POSTN causes up-regulation of vascular endothelial growth factor receptor (VEGFR)-2 in endothelial cells [52]. Elevated expression of VEGFs, the ligands for the VEGF receptors, as observed in some breast carcinomas as well as in our study, provides synergistic paracrine signalling through VEGFR-2 on endothelial cells, potentially promoting angiogenesis and dissemination. Although the expression of POSTN shows a weak correlation with Ki67 immunoreactivity, there is no evidence to suggest that POSTN itself influences proliferation or is a surrogate marker of proliferation rate. Rather, it seems more likely that that its prognostic significance may be due to the altered therapeutic responses of POSTN positive tumours to drugs like tamoxifen. The fact that tumour-specific expression of VEGFR-2 has been associated with an impaired response to tamoxifen therapy in ER-positive premenopausal breast cancer [55] is in line with the poor prognosis of this cohort of breast cancers. Therefore, further studies are required to investigate if POSTN positivity is correlated with VEGFR-2 expression, thereby providing a molecular mechanism that links POSTN to endocrine resistance for ER-positive breast tumours.

Metastasis to bone occurs frequently in advanced breast cancer and is accompanied by debilitating skeletal complications [56]. Among the up-regulated gene sets in the malignant sample with enrichment in myoepithelial/basal type genes in this study was a small family of genes involved in bone remodelling and skeletal development. Their expression in the human breast epithelial cells, including the normal myoepithelial cells, indicates that they play a significant role in epithelial cell biology, in addition to mesenchymal development. Many of these mesenchymal-specific genes, associated with osteoblasts, have previously been found overexpressed in other primary breast tumours [57]. By acquiring the expression of such mesenchymal genes, the malignant epithelial breast cells may have an advantage in growth in the bone environment correlating with progression into a more aggressive cancer phenotype. Targeting such genes and proteins might, therefore, be a means of suppressing this phenomenon.

## Conclusion

In the past decade, several different expression and proteomics studies on purified cell populations of normal luminal and myoepithelial, as well as tumour enriched cell populations, have been carried out [11-13,58,59]. Genes characterising these cell types have been identified, some of which showed altered expression levels in the malignant compared to the normal breast epithelium. In this study, we have taken this profiling forward by comprehensively defining the transcriptomes of highly enriched normal and malignant breast epithelial cell populations on a genome wide scale using multiple technologies. We present here, for the first time, co-regulated breast tumour-associated gene sets enriched in either luminal or myoepithelial-type genes. These data are important for evaluating the breast cancer stratification systems based on established expression profiling, in which luminal and basal phenotypes have been shown to be prognostically significant. Further analysis of these related gene subsets, including expression studies in individual tumours, will assist in our understanding of the mechanisms involved in the initiation and progression of breast cancer, and the loss or acquisition of luminal or myoepithelial phenotypes in breast tumours. This will lead to the identification of additional luminal and basal markers and targets, with importance in the biology of breast cancer and its treatment.

## Abbreviations

COMP = cartilage oligomeric matrix protein; DTET = differential tumour epithelial transcriptome; ER = estrogen receptor; GO = Gene Ontology; GSEA = gene set enrichment analysis; HTR = human transcriptome database; IL = interleukin; MAPK = mitogen-activated protein kinases; MIAME = minimum information about a microarray experiment; MPSS = massively parallel signature sequencing; POSTN = periostin; PR = progesterone receptor; RT-PCR = reverse transcription PCR; SAGE = serial analysis of gene expression; TMA = tissue microarray; tpm = transcripts per million; VEGF = vascular endothelial growth factor; VEGFR = vascular endothelial growth factor receptor.

## Competing interests

The authors declare that they have no competing interests.

## Authors' contributions

This study was conceived by AG, AM and MJOH. The expression profiling and statistical analysis was carried out by AG, AM, KF and MI. The pathological analysis and immunohistochemistry were performed by JSRF and DS. ML performed the RT-PCRs. CI, BS, HV and CVJ participated in the sequence alignment and annotation of MPSS data. The manuscript was written by AG, AM, AMN and MJOH with help from JRSF, PSJ, AA, AJGS and RLS. All authors read and approved the final manuscript. AG and AM contributed equally to this work.

## Supplementary Material

Additional file 1 A jpeg figure showing cell separation of normal and malignant breast epithelial cells. Purity of separated normal and malignant cells. **(a) **A short-term primary culture of breast epithelium stained with monoclonal antibodies specific for vimentin (green), CK 14 (red), CK 18 (blue) and CK 19 (purple), as visualised with appropriate class and sub-class specific fluorescence conjugated secondary antibodies (×150). The middle and right columns show the double immunomagneticallly separated luminal and myoepithelial preparations stained in the same manner, illustrating their homogeneity in respect of cells expressing luminal (CK 18/CK 19) and myoepithelial markers (CK 14/vimentin). **(b) **The irregular clusters of cohesive malignant epithelial cells obtained when a disaggregated tumour is subject to filtration, sedimentation and negative selection for fibroblast activation protein-positive reactive stromal cells and visualised by phase-contrast microscopy to identify samples with minimal microvessel and lymphocytic contamination (×400). Click here for file

Additional file 2 A Word document showing the pathology of primary breast tumours used for MPSS and microarray analysis. The pathological information of 15 primary breast tumours regarding grade, type, size of vascular invasion, lymph node status, estrogen (ER), progesteron (PR) and Her-2 status is provided. Click here for file

Additional file 3 A Word document providing a detailed description of the tissue microarray. Summary of clinicopathological features of patients included in the tissue microarrays. A cohort of 245 invasive breast carcinomas from 245 patients treated with surgery (wide local excision or mastectomy) and adjuvant anthracycline-based chemotherapy was retrieved from the Department of Histopathology files of the Royal Marsden Hospital with appropriate local Ethical Committee approval (Royal Marsden Hospital, London, UK). Representative blocks from 245 invasive breast carcinomas were reviewed by a pathologist (JSRF) and included in duplicate in two tissue microarray (TMA) blocks as previously described. In brief, 0.6 mm core tissue specimens were taken from selected areas of donor blocks (original tumour blocks) and precisely arrayed into two new recipient paraffin blocks (20 × 35 mm) with a custom-built precision instrument (Beecher Instruments, Silver Spring, MD, USA). The presence of tumour tissue in the arrayed sample was verified on a haematoxylin and eosin stained section. ER, PR, p53, vascular invasion, Ki67 (MIB-1) labelling index and nodal status were known for all samples. Follow up was available for 244 patients, ranging from 0.5 to 135.3 months (median = 67.3 months, mean = 67.3 months). Click here for file

Additional file 4 An Excel file listing raw and annotated MPSS data for malignant and normal breast epithelial samples. Sequence signatures with their corresponding annotation and their expression in tpm are shown. Transcripts uniquely expressed in the malignant breast epithelium and in the normal luminal epithelium are highlighted in yellow and red, respectively. Click here for file

Additional file 5 An Excel file containing a table showing an overlay of the multiple microarray platforms based on the HTR database. Microarray featuers of Affymetrix U133 Plus 2.0 GeneChip and CodeLink™ Human Whole Genome Bioarray, Agilent Whole Human Genome Oligo Microarray 44 k cDNA array and 20 k brk cDNA microarray were mapped onto the HTR database. Click here for file

Additional file 6 An Excel file containing a table showing the semi-quantitative RT-PCR of transcripts belonging to the three groups (MPSS-only, MPSS-array confirmed and Array-only). Transcripts with their respective annotation, RT-PCR primer sequence and level of expression detected by RT-PCR are shown. Click here for file

Additional file 7 An Excel file containing a table showing the differential tumour epithelial transcriptome. All 8,051 differentially expressed normal luminal versus tumour genes are listed with their HTR cluster_ID, microarray_ID and their respective fold change for each microarray platform, comprising the differentially expressed epithelial tumour transcriptome. Click here for file

Additional file 8 An Excel file containing a table showing the biological processes deregulated in the DTET. Biological processes of the up- and down-regulated transcripts are shown. Gene Ontology identifiers, description, total number of input genes, as well as *P *value are shown. The input genes for the most significant deregulated biological processes are provided by their gene names and their RefSeq accession numbers. Click here for file

Additional file 9 An Excel file containing a table showing the differential normal epithelial transcriptome. Luminal and myoepithelial transcriptomes based on multiple microarray analyses HTR cluster, microarray feature, fold change and *P *value are listed for each gene. Click here for file

Additional file 10 A Word document detailing the univariate analysis of POSTN on the tumour tissue microarray. Univariate analysis of clinicopathological and immunohistochemical data on the 245 tumour tissue microarray with respect to the epithelial expression of POSTN. *P *values were calculated by the log-rank test. Click here for file

Additional file 11 Cumulative Kaplan-Meier curves for epithelial expression of POSTN. Probability of **(a) **overall survival (OS) and **(b) **disease free survival (DFS) for all patients; and probability of **(c) **OS and **(d) **DFS for only patients with ER positive tumours. *P *values were calculated by the log-rank test. Click here for file

## Figures and Tables

**Table 1:** Numerical analysis of massively parallel signature sequencing

	Malignant breast epithelium	Normal luminal epithelium
Sequence signatures	24,288	28,404
Uniquely mapped signatures	14,245	10,249
Unique HTR clusters	8,421 (3,191)^a^	6,477 (1,297)^a^
Dynamic range	<9,808 tpm	<35,847 tpm
Differentially expressed transcripts	4,311 T > L	2,242 L > T

Sequence signatures represent the total number of sequences obtained by massively parallel signature sequencing (MPSS). Uniquely mapped signatures correspond to the total number of human transcriptome clusters identified and retained in the 'gene-centric' annotation. Unique human transcriptome database (HTR) clusters are transcripts that mapped to a single human cluster and had an abundance of ≥3 transcripts per million (tpm) (approximately one transcript/cell). As described in Materials and methods, statistically significantly (*P *≤ 0.05) differentially expressed transcripts were determined and separated into tumour (T) over normal luminal (L) or vice verse, depending on their fold change. ^a^Corresponds to HTR clusters found in only one sample.

**Table 2:** Multivariant proportional-hazard analysis

Parameter	Hazard ratio (95% confidence interval)	*P *(Cox)
*Grade*	*2.6434 (1.3935–5.0144)*	*0.0029*
*LN status*	*3.6408 (1.5264–8.6840)*	*0.0036*
*POSTN*	*1.8099 (0.9247–3.5422)*	*0.0833*
**LN status**	**5.6924 (1.3331–24.3076)**	**0.0189**
**POSTN**	**2.8151 (1.2048–6.5775)**	**0.0168**

The tissue microarray cohort was analysed using the Cox proportional hazards model for disease-free survival (italic) and overall survival (bold). Only those statistically significant independent prognostic factors as determined by the model are shown. LN, lymph node status at diagnosis.
