# Supplementary figures and images for: Establishment of the epithelial-specific transcriptome of normal and malignant human breast cells based on MPSS and array expression data
Source: Breast Cancer Res. 2006 Oct 2;8(5):R56. doi: 10.1186/bcr1604 (PMC1779497; doi:10.1186/bcr1604)

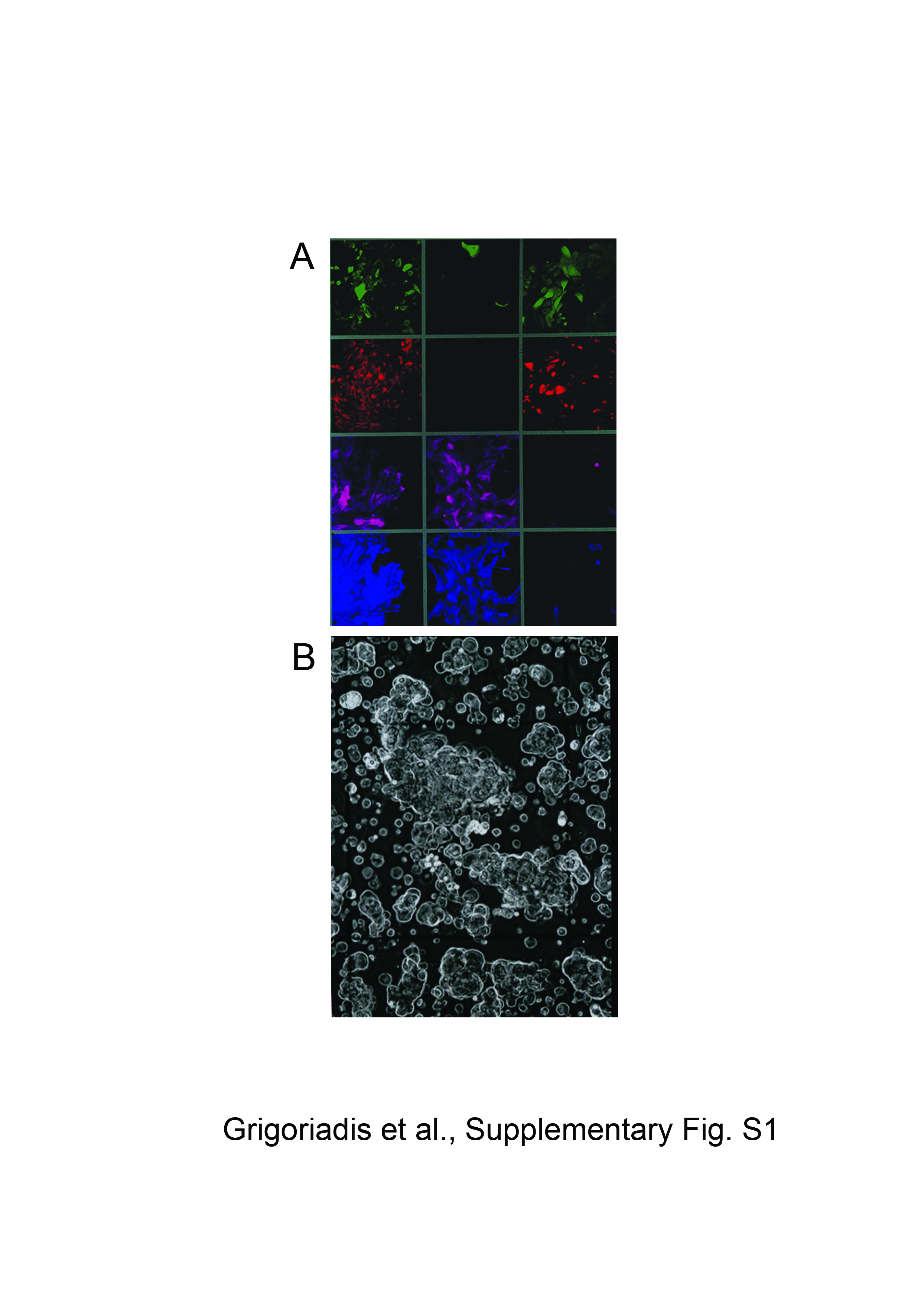

Supplement: Additional file 1 — A jpeg figure showing cell separation of normal and malignant breast epithelial cells. Purity of separated normal and malignant cells. (a) A short-term primary culture of breast epithelium stained with monoclonal antibodies specific for vimentin (green), CK 14 (red), CK 18 (blue) and CK 19 (purple), as visualised with appropriate class and sub-class specific fluorescence conjugated secondary antibodies (×150). The middle and right columns show the double immunomagneticallly separated luminal and myoepithelial preparations stained in the same manner, illustrating their homogeneity in respect of cells expressing luminal (CK 18/CK 19) and myoepithelial markers (CK 14/vimentin). (b) The irregular clusters of cohesive malignant epithelial cells obtained when a disaggregated tumour is subject to filtration, sedimentation and negative selection for fibroblast activation protein-positive reactive stromal cells and visualised by phase-contrast microscopy to identify samples with minimal microvessel and lymphocytic contamination (×400). [file bcr1604-S1.jpeg]
