# Supplementary material for: Establishment of the epithelial-specific transcriptome of normal and malignant human breast cells based on MPSS and array expression data
Source: Breast Cancer Res. 2006 Oct 2;8(5):R56. doi: 10.1186/bcr1604 (PMC1779497; doi:10.1186/bcr1604)
Supplement: Additional file 2 — A Word document showing the pathology of primary breast tumours used for MPSS and microarray analysis. The pathological information of 15 primary breast tumours regarding grade, type, size of vascular invasion, lymph node status, estrogen (ER), progesteron (PR) and Her-2 status is provided. [file bcr1604-S2.doc]

**Supplementary Table S1**. Pathology of individual primary breast tumours used for the malignant pool by MPSS and microarray analysis

| Cell ID | **in situ** | **Grad** | **Type** | **Size** | **Excised** | **Vascular Invasion** | **Lymph Node** | **ER** | **PR** | **Her-2** |
| --- | --- | --- | --- | --- | --- | --- | --- | --- | --- | --- |
| 489 | n/a | 3 | IDC | 25 | YES | + | n/a | + | n/a | - |
| 552 | n/a | 3 | IDC | 40 | YES | + | 8/9 | - | n/a | - |
| 593 | LCIS | 2 | ILC | 35 | YES | - | 1/9 | + | n/a | n/a |
| 695 | LICS | 1 | ILC | 42 | YES | - | 3/5 | + | - | n/a |
| 725 | DCIS, high grade | 3 | IDC | 35 | YES | + | 1/15 | + | n/a | n/a |
| 842 | LCIS,DCIS | 3 | IDC | n/a | n/a | + | 1/10 | + | + | - |
| 845 | DICS,high grade | 3 | IDC | 50 | YES | + | 2/9 | + | - | n/a |
| 896 | DCIS,high grade | n/a | n/a | 45 | NO | - | 0/2 | n/a | n/a | n/a |
| 983 | n/a | 3 | IDC | 15 | YES | + | n/a | - | - | + |
| 1000 | n/a | n/a | n/a | n/a | n/a | n/a | n/a | n/a | n/a | n/a |
| 1033 | n/a | 3 | IDC | 58 | YES | - | 1/15 | - | - | - |
| 1045 | DCIS | 2 | IDC | 50 | YES | - | 0/19 | - | n/a | - |
| 1052 | n/a | 3 | IDC | 35 | YES | - | 0/19 | - | - | n/a |
| 1062 | n/a | 2 | IDC | 20 | YES | + | 1/25 | + | - | - |
| 1063 | n/a | 3 | IDC | 45 | YES | + | n/a | + | + | + |
