# Supplementary material for: Establishment of the epithelial-specific transcriptome of normal and malignant human breast cells based on MPSS and array expression data
Source: Breast Cancer Res. 2006 Oct 2;8(5):R56. doi: 10.1186/bcr1604 (PMC1779497; doi:10.1186/bcr1604)
Supplement: Additional file 3 — A Word document providing a detailed description of the tissue microarray. Summary of clinicopathological features of patients included in the tissue microarrays. A cohort of 245 invasive breast carcinomas from 245 patients treated with surgery (wide local excision or mastectomy) and adjuvant anthracycline-based chemotherapy was retrieved from the Department of Histopathology files of the Royal Marsden Hospital with appropriate local Ethical Committee approval (Royal Marsden Hospital, London, UK). Representative blocks from 245 invasive breast carcinomas were reviewed by a pathologist (JSRF) and included in duplicate in two tissue microarray (TMA) blocks as previously described. In brief, 0.6 mm core tissue specimens were taken from selected areas of donor blocks (original tumour blocks) and precisely arrayed into two new recipient paraffin blocks (20 × 35 mm) with a custom-built precision instrument (Beecher Instruments, Silver Spring, MD, USA). The presence of tumour tissue in the arrayed sample was verified on a haematoxylin and eosin stained section. ER, PR, p53, vascular invasion, Ki67 (MIB-1) labelling index and nodal status were known for all samples. Follow up was available for 244 patients, ranging from 0.5 to 135.3 months (median = 67.3 months, mean = 67.3 months). [file bcr1604-S3.doc]

**Supplementary Table S2. Description of tissue microarray.**

Summary of clinicopathological features of patients included in the tissue microarrays. A cohort of 245 invasive breast carcinomas from 245 patients treated with surgery (wide local excision or mastectomy) and adjuvant anthracycline-based chemotherapy was retrieved from the Department of Histopathology files of the Royal Marsden Hospital with appropriate local Ethical Committee approval (Royal Marsden Hospital, London, UK). Representative blocks from 245 invasive breast carcinomas were reviewed by a pathologist (J.S.R.F.) and included in duplicate in two tissue microarray (TMA) blocks as previously described. In brief, 0.6mm core tissue specimens were taken from selected areas of donor blocks (ie, original tumour blocks) and precisely arrayed into two new recipient paraffin blocks (20 x 35 mm) with a custom-built precision instrument (Beecher Instruments, Silver Spring, MD, USA). The presence of tumour tissue in the arrayed sample was verified on a haematoxylin and eosin stained (H&E)-section. ER, PR, p53, vascular invasion, MIB-1 labelling index and nodal status were known for all samples. Follow up was available for 244 patients, ranging from 0.5 to 135.3 months (median=67.3 months, mean=67.3 months).

|  |  |  |
| --- | --- | --- |
| Parameter |  |  |
| Age (years) |  | 18 – 75 (Median=48, Mean=48) |
| Tumour size (cm) |  | 0.4 – 12 (Median=20, Mean=22) |
| Type | Ductal | 185 |
|  | Lobular | 27 |
|  | Mixed | 25 |
|  | Special types | 8 |
|  |  |  |
| Grade | I | 23 |
|  | II | 69 |
|  | III | 148 |
|  | NA | 5 |
|  |  |  |
| Vascular invasion | Absent | 82 |
|  | Present | 161 |
|  | NA | 2 |
|  |  |  |
| Lymph node metastasis | Absent | 83 |
|  | Present | 154 |
|  | NA | 8 |
|  |  |  |
| Surgical treatment | Mastectomy | 69 |
|  | WLE | 155 |
|  | Others | 20 |
|  |  |  |
| Adjuvant Chemotherapy | FEC | 198 |
|  | FAC | 22 |
|  | ECisF | 17 |
|  | AC | 4 |
|  | ECarboF | 3 |
|  |  |  |
| Hormone treatment | No | 24 |
|  | Tamoxifen | 212 |
|  | Anastrazole | 3 |
|  | Letrozole | 2 |
|  | Tamoxifen+Letrozole | 3 |
|  |  |  |
| Radiotherapy | No | 28 |
|  | Yes | 216 |
|  |  |  |
| Oestrogen receptor | Negative | 198 |
|  | Positive | 48 |
|  | NA | 5 |
|  |  |  |
| Progesterone receptor | Negative | 65 |
|  | Positive | 175 |
|  | NA | 5 |
|  |  |  |
| HER2 | Negative | 205 |
|  | Positive (3+) | 32 |
|  | NA | 8 |
|  |  |  |
| EGFR | Negative | 22 |
|  | Positive | 223 |
|  |  |  |
| Basal keratins | Negative | 208 |
|  | Positive | 35 |
|  | NA | 2 |
|  |  |  |
| p53 | Negative | 159 |
|  | Positive | 67 |
|  | NA | 19 |
|  |  |  |
| MIB-1 | Low (<10%) | 97 |
|  | Intermediate (10-30%) | 97 |
|  | High (>30%) | 33 |
|  | NA | 18 |
|  |  |  |
