# Supplementary material for: Establishment of the epithelial-specific transcriptome of normal and malignant human breast cells based on MPSS and array expression data
Source: Breast Cancer Res. 2006 Oct 2;8(5):R56. doi: 10.1186/bcr1604 (PMC1779497; doi:10.1186/bcr1604)
Supplement: Additional file 10 — A Word document detailing the univariate analysis of POSTN on the tumour tissue microarray. Univariate analysis of clinicopathological and immunohistochemical data on the 245 tumour tissue microarray with respect to the epithelial expression of POSTN. P values were calculated by the log-rank test. [file bcr1604-S10.doc]

**Univariate analysis of POSTN on 245 tumour tissue microarray.**

| Parameter |  | No. available | Not assessable | POSTN + | POSTN - | Statistical association |
| --- | --- | --- | --- | --- | --- | --- |
| Grade | 1 | 224 | 21 | 6 | 15 | p=0.06 |
|  | 2 |  |  | 6 | 57 | Chi-square test |
|  | 3 |  |  | 30 | 110 |  |
|  |  |  |  |  |  |  |
| LVI | + | 226 | 19 | 32 | 116 | p=0.1494 |
|  | - |  |  | 10 | 68 | Fisher’s exact test |
|  |  |  |  |  |  |  |
| LN mets | + | 221 | 24 | 29 | 113 | p=0.3719 |
|  | - |  |  | 12 | 67 | Fisher’s exact test |
|  |  |  |  |  |  |  |
| ER | + | 227 | 18 | 37 | 142 | p=0.1420 |
|  | - |  |  | 5 | 43 | Fisher’s exact test |
|  |  |  |  |  |  |  |
| PgR | + | 227 | 18 | 36 | 129 | p<0.05 |
|  | - |  |  | 6 | 56 | Fisher’s exact test |
|  |  |  |  |  |  |  |
| HER2 | + | 227 | 18 | 3 | 29 | p=0.2187 |
|  | - |  |  | 39 | 156 | Fisher’s exact test |
|  |  |  |  |  |  |  |
| EGFR | + | 228 | 17 | 4 | 18 | p>0.9999 |
|  | - |  |  | 38 | 168 | Fisher’s exact test |
|  |  |  |  |  |  |  |
| CK 14 | + | 226 | 19 | 1 | 20 | p=0.1368 |
|  | - |  |  | 41 | 164 | Fisher’s exact test |
|  |  |  |  |  |  |  |
| CK 5/6 | + | 218 | 27 | 1 | 24 | p=0.0544 |
|  | - |  |  | 39 | 154 | Fisher’s exact test |
|  |  |  |  |  |  |  |
| CK 17 | + | 225 | 20 | 2 | 25 | p=0.1819 |
|  | - |  |  | 39 | 159 | Fisher’s exact test |
|  |  |  |  |  |  |  |
| Basal markers | - | 226 | 19 | 3 | 35 | p=0.0695 |
|  | + |  |  | 39 | 149 | Fisher’s exact test |
|  |  |  |  |  |  |  |
| Nielsen groups | Basal | 218 | 27 | 3 | 27 | p=0.0771 |
|  | Luminal |  |  | 36 | 120 | Chi-square test |
|  | HER2 |  |  | 3 | 29 |  |
|  |  |  |  |  |  |  |
| P53 | + | 221 | 24 | 13 | 52 | p=0.7081 |
|  | - |  |  | 28 | 128 | Fisher’s exact test |
|  |  |  |  |  |  |  |
| Ki67(MIB-1) | <10% | 222 | 23 | 14 | 80 | p<0.05 |
|  | 10-30% |  |  | 25 | 71 | Chi-square test |
|  | >30% |  |  | 3 | 29 |  |
|  |  |  |  |  |  |  |
| CCND1 amp | Amp | 226 | 19 | 1 | 27 | p<0.05 |
|  | No amp |  |  | 41 | 157 | Fisher’s exact test |
|  |  |  |  |  |  |  |
| COMP | + |  |  | 14 | 18 | p<0.0005 |
|  | - |  |  | 26 | 167 |  |

LVI (lymph node invasion), LN met Lymph node metastases.
